# Oxygen concentration modulates HDAC1-Mediated regulation of osteogenic signaling pathways in dental pulp cells

**DOI:** 10.3389/fcell.2025.1627763

**Published:** 2025-09-17

**Authors:** Ci Song, Ping Li, Lin Lin, Ge Cao, Zhao Liu, Fei Liu, Ling Peng, Jingxing Dai, Buling Wu, Ting Chen

**Affiliations:** ^1^ Department of Stomatology, Nanfang Hospital, Southern Medical University, Guangzhou, China; ^2^ College of Stomatology, Southern Medical University, Guangzhou, China; ^3^ Department of Stomatology, The Eighth Medical Center of PLA General Hospital, Beijing, China; ^4^ Guangdong Provincial Key Laboratory of Digital Medicine and Biomechanics & Guangdong Engineering Research Center for Translation of Medical 3D Printing Application & National Virtual & Reality Experimental Education Center for Medical Morphology (Southern Medical University) & National Experimental Education Demonstration Center for Basic Medical Sciences (Southern Medical University) & National Key Discipline of Human Anatomy, School of Basic Medical Sciences, Southern Medical University, Guangzhou, China

**Keywords:** hypoxic microenvironment, dental pulp stem cells, osteogenic differentiation, regenerative endodontics, epigenetic regulation

## Abstract

**Background:**

Dental pulp regeneration represents a critical frontier in translational dentistry, with dental pulp stem cells (DPSCs) demonstrating exceptional reparative potential through their multipotent differentiation capacity. While oxygen tension is known to influence cellular physiology, its regulatory mechanisms on DPSC osteo/odontogenic differentiation remain poorly understood.

**Methods:**

We established physiologically relevant oxygen gradients (3%, 5%, 21% O_2_) to mimic developmental and pathological pulp microenvironments. Cellular proliferation and osteogenic capacity were assessed through flow cytometry, CCK-8 assays, and Live/Dead staining. Differentiation markers (RUNX2, OCN, ALP, DSPP) were quantified via qRT-PCR, immunoblotting, and enzymatic activity assays. Pharmacological inhibition studies using Oltipraz (HIF-1α inhibitor) and Valproic acid (HDAC inhibitor) elucidated pathway interactions. Publicly available transcriptomic datasets were analyzed to identify hypoxia-regulated pathways, and protein interactions were predicted using bioinformatics tools.

**Results:**

Moderate hypoxia (5% O_2_) significantly enhanced DPSC proliferation (p < 0.05 vs. normoxia) and upregulated osteogenic markers at transcriptional (1.8–3.2 fold) and translational levels. Severe hypoxia (3% O_2_) suppressed both proliferation (p < 0.01) and differentiation markers (0.4–0.7 fold). HIF-1α inhibition reversed 5% O_2_-mediated osteogenic enhancement (p < 0.01), while HDAC1 blockade with Valproic acid rescued differentiation capacity under 3% O_2_ (1.5–2.1 fold induction). Mechanistically, HDAC1 appeared to influence HIF-1α protein levels in an oxygen-dependent manner, and its inhibition affected pathways consistent with alterations in chromatin remodeling, influencing VEGFA-mediated osteogenic signaling.

**Conclusion:**

Our findings establish an oxygen-sensitive HDAC/HIF-1α regulatory axis governing DPSC fate determination. The biphasic response to hypoxia gradients suggests microenvironmental optimization strategies could enhance pulp regenerative outcomes. These insights provide mechanistic foundations for developing HDAC-targeted approaches in endodontic regeneration.

## Introduction

Pulpitis and apical periodontitis represent the most prevalent dental pathologies necessitating endodontic intervention (Oguntebi and Shen, 1992). Conventional root canal therapy, while effective in disease containment, results in devitalized teeth prone to structural compromise (Caplan et al., 2005). This clinical challenge has driven interest in pulp regeneration strategies leveraging the innate plasticity of dental pulp stem cells (DPSCs) (Gronthos et al., 2000; Huang et al., 2009; Mitsiadis et al., 2011). Despite successful DPSC-mediated dentin-pulp complex regeneration in preclinical models (Xuan et al., 2018), translational applications remain limited by suboptimal cell survival and differentiation control in implantation settings (Li et al., 2020).

Oxygen tension constitutes a critical niche parameter regulating stem cell fate, with physiological pulp oxygen levels reported to range approximately from 3%–6% depending on vascularization and metabolic state, while pathological or inflammatory conditions can further alter these levels (Kazmi et al., 2013; Patel et al., 2022; van Santen et al., 2022). Thus, understanding cellular responses within this range is crucial. Standard *in vitro* culture at atmospheric oxygen (21% O_2_) induces oxidative stress and inflammatory responses (Ciccone et al., 2013), highlighting the need for physiomimetic models. Current literature presents conflicting data on hypoxic effects: while 3% O_2_ promotes DPSC angiogenic potential (Liu et al., 2019), its impact on osteo/odontogenesis remains controversial with reports of both enhanced (Ito et al., 2015) and suppressed differentiation (Iida et al., 2010; Zayed et al., 2021). This discrepancy underscores the need for systematic evaluation across physiologically relevant oxygen gradients.

Emerging evidence implicates epigenetic regulation through histone deacetylases (HDACs) in hypoxia adaptation (Ito et al., 2015). Our previous work identified HDAC1 as a key mediator of oxidative stress responses in DPSCs under 3% O_2_ (Liu et al., 2019), suggesting potential crosstalk with hypoxia-inducible factor 1α (HIF-1α) signaling. However, the oxygen-dependent interplay between HDAC activity (potentially influencing chromatin remodeling) and osteogenic pathway activation remains to be fully elucidated. Therefore, this study aimed to investigate the effects of different physiologically relevant hypoxic concentrations (3% and 5% O_2_) on DPSC proliferation and osteo/odontogenic differentiation, and to explore the potential regulatory roles of the HDAC1/HIF-1α axis in this process by examining the expression of key molecules and the effects of their pharmacological inhibition.

## Materials and methods

### Isolation and culture of human DPCs

Primary human dental pulp cells (hDPCs) were isolated from intact third molars extracted from healthy adults (19–25 years) at the Department of Oral and Maxillofacial Surgery, Nanfang Hospital, Southern Medical University. The study protocol was approved by the Institutional Review Board of Nanfang Hospital (Approval No. NFH-2023-DPSC01), with written informed consent obtained from all donors. Experiments were performed in triplicate and repeated with cells derived from at least three different healthy donors to account for biological variability.

Pulp tissues were dissected under sterile conditions following established protocols (Gronthos et al., 2000). Briefly, teeth were disinfected with phosphate-buffered saline (PBS) and sectioned at the cementoenamel junction. Excised pulp tissues were digested in 3 mg/mL type I collagenase (Sigma-Aldrich) for 15 min at 37 °C, then cultured in Dulbecco’s Modified Eagle Medium (DMEM; Gibco) supplemented with 10% fetal bovine serum (FBS; HyClone), 100 U/mL penicillin, and 100 μg/mL streptomycin (HyClone). Medium was replaced after 24 h and subsequently every 72 h. Cells from passages 3-5 were used for experiments to ensure phenotypic stability.

### Hypoxic microenvironment treatment

Cells were exposed to defined oxygen tensions using an Anoxomat Mark II system (Advanced Instruments). Experimental groups included:

1. Severe hypoxia (3% O_2_)
2. Moderate hypoxia (5% O_2_)
3. Normoxic control (21% O_2_)

These oxygen concentrations were chosen to represent a range from severe physiological/pathological hypoxia (3% O_2_) to moderate physiological hypoxia (5% O_2_), compared with standard atmospheric culture conditions (21% O_2_). All conditions maintained 5% CO_2_ at 37 °C with humidity >95%. Gas concentrations were verified daily using a built-in optical sensor.

### Flow cytometry analysis

Cell cycle distribution was assessed using propidium iodide (PI) staining. Briefly, 5 × 10^5^ cells per condition were fixed in 70% ice-cold ethanol for ≥12 h at 4 °C. Samples were treated with 150 μL RNase A (100 μg/mL) for 30 min at 37 °C, followed by 150 μL PI staining solution (50 μg/mL) for 30 min at 4 °C protected from light. DNA content was analyzed using a BD FACSCanto II flow cytometer with ModFit LT software (Verity Software House).

### Cell proliferation assay

Proliferation kinetics were determined using CCK-8 (Dojindo, JE603). Cells (2 × 10^3^/well) were seeded in 96-well plates and cultured under respective oxygen conditions for 1, 3, 5, 7, and 9 days. Absorbance at 450 nm was measured 2 h after CCK-8 reagent addition using a SpectraMax M5 microplate reader (Molecular Devices).

### Cell viability assay

Live/Dead staining (Abcam, ab115347) was performed per manufacturer’s instructions (Zhan et al., 2020). After 48 h culture, cells were incubated with 2 μM calcein-AM and 4 μM ethidium homodimer-1 for 30 min at 37 °C. Viability was quantified using fluorescence microscopy (Nikon Eclipse Ti2) with NIS-Elements software.

### *In Vitro* osteogenic differentiation

Cells (1 × 10^6^/dish) were induced with osteogenic medium containing:

• DMEM +10% FBS
• 50 μg/mL ascorbic acid (Sigma)
• 10 mM β-glycerophosphate (Sigma)
• 100 nM dexamethasone (Sigma)

Medium was refreshed every 3 days under respective oxygen conditions.

### ALP activity assay

After 7-day induction, cells were fixed in 10% neutral buffered formalin for 15 min. Alkaline phosphatase activity was quantified using p-nitrophenyl phosphate substrate (Sigma, 85L-s) per manufacturer’s protocol. Absorbance at 405 nm was normalized to total protein content measured by BCA assay.

### Alizarin Red S staining

Matrix mineralization was assessed after 21-day induction. Cells were fixed in 70% ethanol for 1 h and stained with 2% Alizarin Red S (pH 4.2; Sigma) for 10 min. Calcium deposition was quantified by 10% cetylpyridinium chloride extraction and absorbance measurement at 562 nm.

### Quantitative reverse transcription PCR (RT-qPCR)

Total RNA was extracted using TRIzol (Invitrogen) after 14-day induction. cDNA synthesis employed PrimeScript RT Master Mix (Takara, 0036A). Gene expression was analyzed on a LightCycler 480 II (Roche) with SYBR Green detection. Primer sequences are listed in Table 1. Data were normalized to β-actin using the 2^(-ΔΔCt) method.

**TABLE 1:** Primers used for RT-qPCR.

Gene	Primer
β-actin	F-AGAGCTACGAGCTGCCTGACG R-GGACTCCATGCCCAGGAAGGA
Runx2	F-TGGTTACTGTCATGGCGGGTA R-TCTCAGATCGTTGAACCTTGCTA
ALP	F-GCAACTTCCAGACCATTGGC R-TCCCACTGACTTCCCTGCTT
OCN DSPP	F-AGCCCATTAGTGCTTGTAAAGG R-CCCTCCTGCTTGGACACAAAG F-TGGCGATGCAGGTCACAAT R-CCATTCCCACTAGGACTCCCA
HIF-1α HDAC1	F- GAACGTCGAAAAGAAAAGTCTCG R- CCTTATCAAGATGCGAACTCACA F-CTACTACGACGGGGATGTTGG R-GAGTCATGCGGATTCGGTGAG
HDAC2 HDAC3	F-ATGGCGTACAGTCAAGGAGG R-TGCGGATTCTATGAGGCTTCA F-CCTGGCATTGACCCATAGCC R-CTCTTGGTGAAGCCTTGCATA
HDAC4 HDAC5	F-GGCCCACCcGGAATCTGAAC R-GAACTCTGGTcaaGGGGAACTG F-GGCCCACCGGAATCTGAAC R-GAACTCTGGTCAAGGGAACTG F-TCTTGTCGAAGTCAAAGGAGC R-GAGGGGAACTCTGGTCCAAAG
HDAC6 HDAC7	F-AAGAAGACCTAATCGTGGGACT R-GCTGTGAACCAACATCAGCTC F-GGCGGCCCTAGAAAGAACAG R-CTTGGGCTTATAGCGCAGCTT
HDAC8 HDAC9	F-TCGCTGGTCCCGGTTTATATC R-TACTGGCCCGTTTGGGGAT F-AGTAGAGAGGCATCGCAGAGA R-GGAGTGTCTTTCGTTGCTGAT
HDAC10 HDAC11	F-CAGTTCGACGCCATCTACTTC R-CAAGCCCATTTTGCACAGCTC F-ACCCAGACAGGAGGAACCATA R-TGATGTCCGCATAGGCACAG
Sirt1 Sirt2	F-TAGCCTTGTCAGATAAGGAAGGA R-ACAGCTTCACAGTCAACTTTGT F-TGCGGAACTTATTCTCCCAGA R-GAGAGCGAAAGTCGGGGAT
Sirt3 Sirt4	F-ACCCAGTGGCATTCCAGAC R-GGCTTGGGGTTGTGAAAGAAG F-GCTTTGCGTTGACTTTCAGGT R-CCAATGGAGGCTTTCGAGCA
Sirt5 Sirt6	F-GCCATAGCCGAGTGTGAGAC R-CAACTCCACAAGAGGTACATCG F-GCAGTCTTCCAGTGTGGTGT R-CCAGTTTGTCCCTGGGGAAG
Sirt7	F-GACCTGGTAACGGAGCTGC R-CGACCAAGTATTTGGCGTTCC

### Western blot analysis

Following specified culture periods under different oxygen conditions, with or without osteogenic induction and/or inhibitor treatments, total protein was extracted from DPCs using RIPA lysis buffer (Beyotime, China) containing protease and phosphatase inhibitors. Protein lysates (20 μg/lane) were separated on 10% SDS-PAGE gels and transferred to PVDF membranes. After blocking with 3% BSA, membranes were incubated overnight at 4 °C with primary antibodies:

• RUNX2 (1:1000; Abcam ab192256)
• OCN (1:1000; Cell Signaling 3716)
• DSPP (1:1000; Santa Cruz sc-73632)
• HDAC1 (1:1000; Cell Signaling 2062)
• VEGFA (1:1000; Abcam ab46154)
• HIF-1α (1:500; Abcam ab279654)
• β-actin (1:5000; Millipore MAB1501)

HRP-conjugated secondary antibodies (LI-COR; 1:10,000) were detected using ECL Prime (Pierce). Band intensity was quantified with ImageJ (NIH).

### Pharmacological inhibition studies

To investigate the roles of HDAC1 and HIF-1α, pharmacological inhibitors were used. Based on preliminary cytotoxicity assays (CCK-8, data shown in Figures 5a,b) and literature, the following concentrations were selected:

• HDAC inhibitor: Valproic acid (VPA; MedChemExpress HY-10585; 100 μM)
• HIF-1α inhibitor: Oltipraz (MedChemExpress HY-12519; 10 μM)

Cells were pre-treated with inhibitors for 2 h before exposure to respective oxygen conditions and/or osteogenic induction medium. Inhibitors were replenished with each medium change.

### Bioinformatic analysis of public transcriptomic data and protein interaction prediction

To identify pathways potentially regulated by hypoxia in DPCs, publicly available Gene Expression Omnibus (GEO) datasets (GSE118046 (Liu et al., 2019), GSE45872 (Iida et al., 2013)) were analyzed. These datasets contain transcriptomic data from human dental pulp cells cultured under normoxic and hypoxic conditions. Raw data were processed using Feature Extraction 10.7.1.1 (Agilent) and GeneSpring 14.9 (Agilent) if applicable based on original data format, or standard pipelines for RNA-seq data. Functional enrichment analysis for Gene Ontology (GO) terms and Kyoto Encyclopedia of Genes and Genomes (KEGG) pathways was performed using DAVID 6.8 (Database for Annotation, Visualization and Integrated Discovery) (Huang da et al., 2009), with significance typically determined by p-values <0.05 and FDR correction. Protein-protein interactions were predicted using the STRING database (v11.5) (Szklarczyk et al., 2021) and BioGRID (v4.4) (Oughtred et al., 2021) to explore potential interactions relevant to the study’s focus, such as between HIF-1α and HDAC1.

### Statistical analysis

Data represent mean ± SD of triplicate experiments from at least three independent biological replicates (i.e., cells from different donors), unless otherwise specified. Comparisons used one-way ANOVA with Bonferroni *post hoc* test or Student’s t-test where appropriate, performed using SPSS 19.0 (IBM). Statistical significance was set at P < 0.05. For bioinformatic analyses of public datasets, statistical methods inherent to the tools used (e.g., modified Fisher’s exact test in DAVID, FDR correction) were applied, with significance thresholds typically set at p < 0.05 or as specified in the results.

## Results

### Hypoxic microenvironments enhance cellular proliferation without compromising viability

**FIGURE 1:**
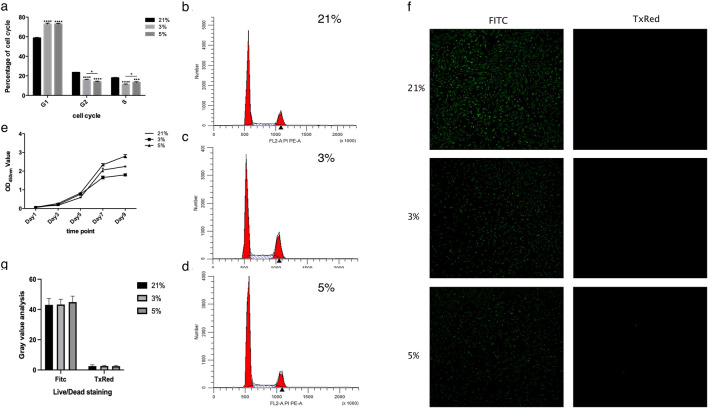
Results of cell cycle, cell proliferation, and cell viability. **(a)** Cell cycle analysis. The proportions of DPCs cultured at 3% and 5% oxygen concentrations in each phase differed significantly from those at 21%. Significant differences were observed specifically in the S and G2/M phases (G1: Gap 1 phase; S: Synthesis phase; G2/M: Gap 2/Mitosis phase). **(b)** Representative cell cycle histogram at 21% oxygen concentration. **(c)** Representative cell cycle histogram at 3% oxygen concentration. **(d)** Representative cell cycle histogram at 5% oxygen concentration. **(e)** Cell proliferation trends of DPCs, as detected by CCK8 assay. **(f)** Representative fluorescence micrographs of Live/Dead staining of cells (Green: Calcein-AM stained live cells; Red: Ethidium homodimer-1 stained dead cells). **(g)** Gray value analysis of Live/Dead staining, showing no significant difference among the three groups. *P < 0.05, ***P < 0.001, ****P < 0.0001. OD, optical density.

Analysis of cell cycle distribution revealed that all experimental groups exhibited a predominant proportion of cells in the G1 phase (48-h exposure), followed by S and G2/M phases (Figures 1a–d). Hypoxic treatment (both 3% and 5% O_2_) increased the S/G2/M phase ratio compared to normoxia, suggesting G1 phase arrest and slower cell cycle progression—a characteristic consistent with stem cell behavior (Li et al., 2013). Significant differences were observed between hypoxic groups (3% and 5% O_2_) and normoxic controls (21% O_2_) in S/G2/M phase distribution (P < 0.0001), while no notable divergence occurred in G1 phase proportions between 3% and 5% O_2_ groups (Figures 1a–d). Notably, the 3% O_2_ group demonstrated higher proliferative activity as indicated by a higher proportion of cells in S/G2/M phase after 48 h than the 5% O_2_ group, which in turn exceeded the 21% O_2_ group at this early timepoint.

Proliferation kinetics assessed via CCK-8 assays revealed sigmoidal growth curves across all oxygen conditions (Figure 1e). Cells cultured under 5% O_2_ exhibited accelerated proliferation between days 5–7, with reduced rates during days 1–5 and 7–9, reaching plateau phase by day 9. Comparative analysis confirmed superior proliferative capacity in the 5% O_2_ group relative to normoxic controls, while the 3% O_2_ group displayed attenuated growth (Figure 1e). Live/Dead staining after 48-h exposure demonstrated comparable viability (>95%) across all oxygen concentrations, with no statistically significant intergroup differences (Figures 1f,g).

### Moderate hypoxia (5% O_2_) potentiates odontogenic differentiation

**FIGURE 2:**
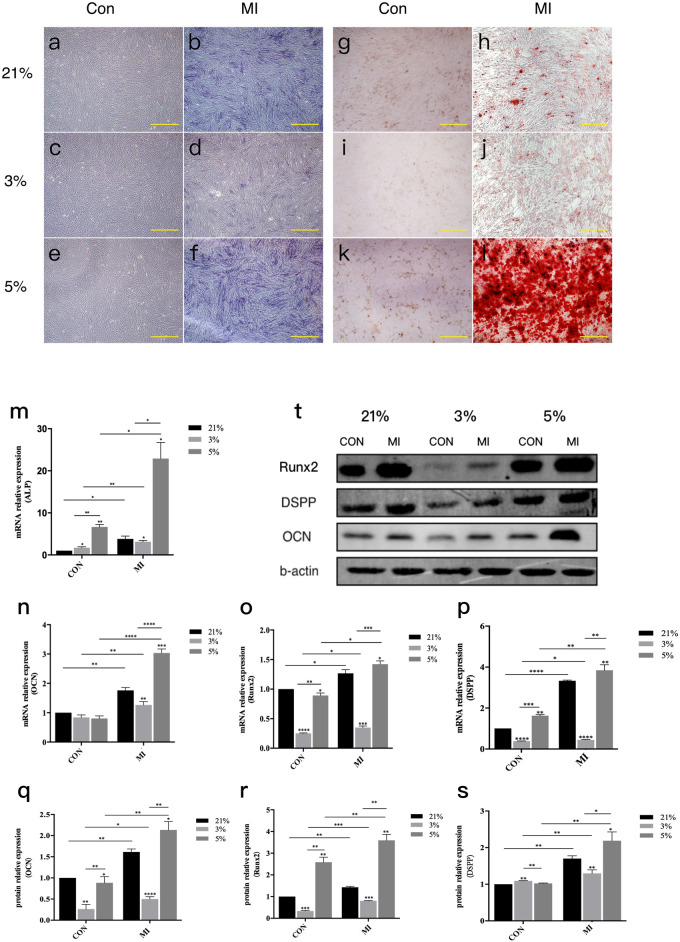
Odontoblast differentiation of DPCs under different oxygen concentrations.ALP activity assay and ARS staining of DPCs in three different microenvironments. **(a–f)** ALP activity assay **(a–c)**: Control groups at 21%, 3%, 5% O_2_ respectively; **(d–f)** Mineralization Inductive (MI) groups at 21%, 3%, 5% O_2_ respectively, showing colorimetric reaction for ALP activity). **(g–l)** ARS staining (**(g–i)**: Control groups at 21%, 3%, 5% O_2_ respectively; **(j–l)** MI groups at 21%, 3%, 5% O_2_ respectively, showing red staining of mineralized nodules). Scale bar, 100 μm. **(m)** mRNA relative expression of ALP. **(n–p)** mRNA relative expression of Ocn, Runx2, and Dspp.**(q)** Representative Western blot images showing protein expression of odontoblast-related factors (RUNX2, DSPP, OCN and β-actin as loading control). **(r,s)** Gray value data for OCN and RUNX2 proteins. **(t)** Protein levels of DSPP (densitometry not shown separately but implied by blot in q). *P < 0.05, **P < 0.01, ***P < 0.001, ****P < 0.0001. Con, control group; MI, mineralized inductive group. Full-length blots are presented in Supplementary Figure S1.

Alkaline phosphatase (ALP) activity assays following 7-day mineralization induction (MI) revealed robust staining in mineralized groups versus non-induced controls (Figures 2a–f). Quantitative analysis demonstrated hierarchical ALP expression: 5% O_2_ MI > 21% O_2_ MI > 3% O_2_ MI (*P* < 0.05). Alizarin Red S (ARS) staining at day 21 corroborated these findings, with 5% O_2_ MI cultures exhibiting significantly greater mineralized nodule density and quantity compared to other groups (Figures 2g–l).

Molecular profiling confirmed oxygen-dependent differentiation patterns. RT-qPCR analysis of 14-day MI cultures showed upregulated mRNA expression of odontogenic markers (*DSPP*, *ALP*, *RUNX2*, *OCN*) in all MI groups versus controls (Figures 2m–p). Comparative quantification revealed:

• 5% O_2_ MI: 1.8–3.2-fold increase relative to 21% O_2_ MI
• 3% O_2_ MI: 0.4–0.7-fold reduction versus 21% O_2_ MI

Western blot analysis mirrored transcriptional patterns, with 5% O_2_ MI inducing marked upregulation of RUNX2, OCN, and DSPP proteins, while 3% O_2_ MI suppressed their expression (Figures 2q–s).

### Oxygen-dependent regulation of hypoxic signaling mediators

**FIGURE 3:**
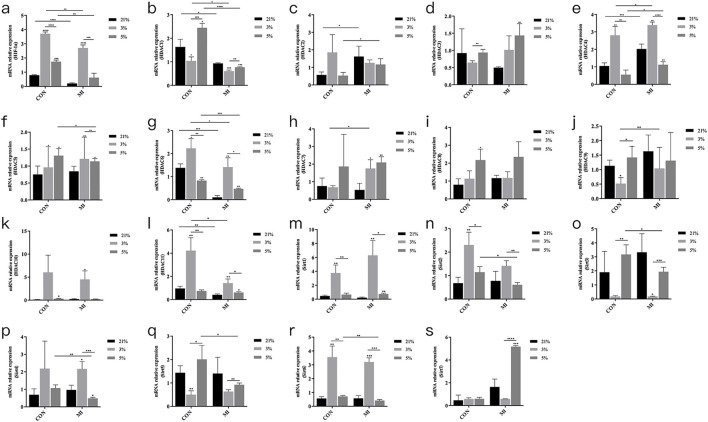
mRNA expression levels of Hif1a and Hdac. **(a–s)** Relative mRNA expression of HIF-1α, HDAC1-11, and Sirt1-7. *P < 0.05, **P < 0.01, ***P < 0.001, ****P < 0.0001. Con, control group; MI, mineralized inductive group.

Hypoxic exposure (24-h) significantly elevated *HIF-1α* mRNA levels in both 3% O_2_ (4.1 ± 0.3-fold) and 5% O_2_ (3.2 ± 0.2-fold) groups versus normoxic controls (*P* < 0.001) (Figure 3a). Extended hypoxic culture (14-day MI) modulated HDAC isoform expression, with *HDAC1*, *HDAC4*, and *HDAC6* demonstrating statistically significant oxygen-dependent regulation (Figures 3b,e,g). Other HDAC family members showed no significant differential expression across oxygen conditions (Figures 3c,d,f–s).

### Bioinformatic analysis of public datasets supports roles for HIF-1α and HDAC1 in odontogenesis

**FIGURE 4:**
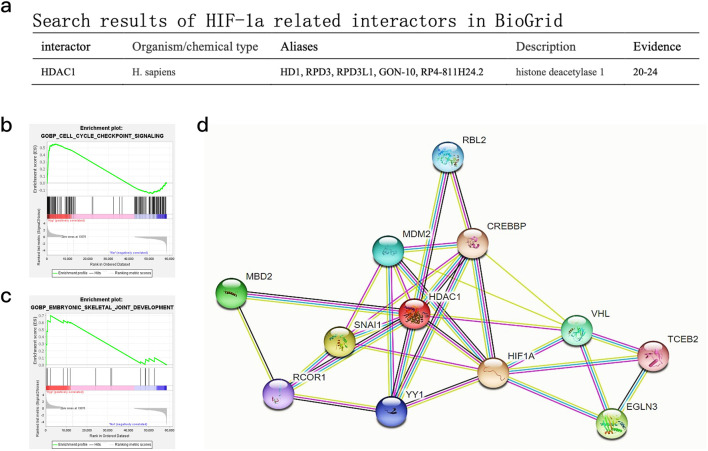
Bioinformatics analysis of publicly available DPC transcriptomic datasets and predicted protein interactions. **(a)** Search results for HIF-1α-related interactors in BioGrid, highlighting HDAC1. **(b)** GSEA pathway enrichment maps for “GOBP_CELL_CYCLE_CHECKPOINT_SIGNALING” based on analysis of public hypoxic DPC datasets. **(c)** GSEA pathway enrichment maps for “GOBP_EMBRYONIC_SKELETAL_JOINT_DEVELOPMENT” based on analysis of public hypoxic DPC datasets. **(d)** Predicted protein interaction maps from STRING database, showing potential interactions centered around HIF1A and HDAC1 with other relevant proteins.

Gene Set Enrichment Analysis (GSEA) of publicly available hypoxia-responsive transcriptomic datasets (GSE118046, GSE45872) (Liu et al., 2019; Iida et al., 2013; Canzler and Hackermuller, 2020) identified significant enrichment (P < 0.0001, FDR <0.0001) of odontogenic differentiation pathways under hypoxic conditions. Of 157 KEGG-annotated odontoblast differentiation genes, 85 showed hypoxia-induced upregulation in these datasets. Network analysis via BioGRID predicted a potential direct interaction between HIF-1α and HDAC1 (Figure 4a), while pathway mapping using these public datasets further revealed HIF-1α-mediated activation of cell cycle and osteogenic signaling cascades (Figures 4b–d).

### Mechanistic interplay between HIF-1α and HDAC1 in odontogenesis

**FIGURE 5:**
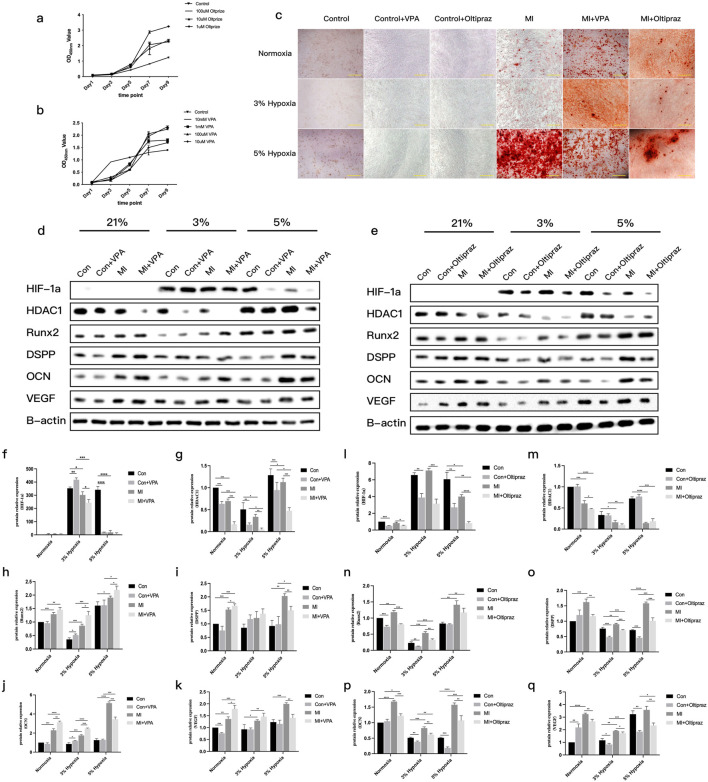
Odontoblast differentiation of DPCs with inhibitors under different oxygen concentrations. **(a)**CCK8 results showing the effect of different concentrations of Oltipraz (HIF-1α inhibitor) on DPC proliferation. **(b)**CCK8 results showing the effect of different concentrations of VPA (HDAC inhibitor) on DPC proliferation. **(c)**ARS staining results of DPCs treated with Oltipraz or VPA in three different microenvironments during mineralization induction. **(d)**Representative Western blot images showing protein levels of HIF-1α, HDAC1, RUNX2, DSPP, OCN, VEGF, and β-actin in different groups with VPA treatment. **(e)**Representative Western blot images showing protein levels of HIF-1α, HDAC1, RUNX2, DSPP, OCN, VEGF, and β-actin in different groups with Oltipraz treatment. **(f–k)**Gray value data for proteins HIF-1α, HDAC1, RUNX2, DSPP, OCN, and VEGF in the VPA-treated groups (Note: original legend had **(f–j)**, now **(f–k)**to match 6 proteins). **(l–q)**Gray value data for proteins HIF-1α, HDAC1, RUNX2, DSPP, OCN, and VEGF in the Oltipraz-treated groups. *P < 0.05, **P < 0.01, ***P < 0.001, ****P < 0.0001. Con, control group; MI, mineralized inductive group. Full-length blots are presented in Supplementary Figure 1.

Pharmacological inhibition studies established 100 μM VPA (HDAC inhibitor) and 10 μM Oltipraz (HIF-1α inhibitor) as non-cytotoxic working concentrations (Figures 5a,b). ARS staining demonstrated oxygen-contextual effects: HDAC inhibition with VPA enhanced differentiation under 21% and 3% O_2_ but attenuated it at 5% O_2_, whereas HIF-1α blockade suppressed differentiation across hypoxic conditions (Figure 5c). Western blot analysis confirmed these phenotypic observations at the protein level (Figure 5d). Intriguingly, HDAC inhibition with VPA reduced HIF-1α protein expression, while HIF-1α blockade did not reciprocally affect HDAC1 levels (Figure 5e). Densitometric quantification of immunoreactive bands validated these regulatory relationships (Figures 5f–q).

## Discussion

This study focused on the role of oxygen concentration in the odontogenic differentiation of DPCs, aiming to provide a theoretical foundation for selecting the optimal oxygen level in the microenvironment. To explore the underlying mechanisms, we screened the HDACs that may be involved in the effects of hypoxia on the odontogenic differentiation of DPCs and investigated the interplay with HIF-1α signaling.

Our findings on cell proliferation showed a nuanced response to hypoxia. While early (48 h) cell cycle analysis suggested increased S/G2/M phases in 3% O_2_ compared to 5% O_2_, longer-term CCK-8 assays indicated that 5% O_2_ ultimately supported superior proliferation compared to 3% O_2_ and normoxia. This contrasts with Ahmed et al. (2016), who reported that 3% hypoxia enhanced DPSC proliferation. However, the study by Werle S showed no significant difference in the proliferation level of SHEDs cultured under 3% oxygen concentration compared to those cultured in a normal oxygen environment (Werle et al., 2019). The discrepancy in conclusions may be attributed to the different cell types used in the experiments (DPCs vs. SHEDs), variations in initial cell status, culture duration, and seeding densities. In this study, DPCs were chosen, while Werle S et al. used SHEDs. The status of pulp stem cells varied, leading to different conclusions. Furthermore, after 14 days of *in vitro* culture, due to cell proliferation, the culture area remained unchanged, resulting in contact between the cells and thus producing a contact inhibition effect, which affected DPC proliferation. In this experiment, the duration of cell culture and inoculation density were considered to assess the impact of cell multiplication (Titova et al., 2023). The proliferation ability of cells is affected by contact inhibition. The results also showed that the proliferation of DPCs followed a slow, periodic pattern, which is consistent with the characteristics of stem cells. After a period of culture, the proliferation level of DPCs may have reached a plateau stage, potentially leading to different experimental outcomes.

Oxygen concentration plays a significant role in the odontogenic differentiation of DPCs (Ma et al., 2023). In our study, DPCs were cultured in a hypoxic microenvironment with different oxygen concentrations. The effects of the hypoxic microenvironment on odontogenic differentiation were further verified by RT-qPCR, Western blot, ALP staining, and ARS staining. ARS staining was performed on DPCs after 21 days of treatment. No obvious mineralized nodules were found in the control groups at 21%, 3%, and 5% oxygen concentrations. In contrast, the corresponding mineralized groups formed prominent reddish-brown mineralized nodules, indicating that DPCs cultured in these oxygen concentrations could differentiate into odontoblasts after mineralization induction. Among the conditions, mineralized nodules cultured under 3% hypoxia were the smallest in both volume and number, while those cultured under 5% exhibited the largest volume and quantity. Compared to the 21% oxygen concentration environment, 5% hypoxia promoted odontogenic differentiation of DPCs, while 3% hypoxia inhibited it. DPCs were able to differentiate into odontoblasts when cultured in a specific induction medium.

To form dentin, the extracellular matrix of odontoblasts is typically deposited with calcium salts. Alizarin Red binds to extracellular calcium salts to form reddish-brown chelates. The depth of color represents the number of mineralized nodules in the cell structure (Tsukamoto et al., 1992). Induced odontogenic differentiation of DPCs *in vitro* showed reddish-brown deposition. The results at 3%, 5%, and 21% oxygen concentrations differed significantly. ARS staining results were notably different. A possible mechanism is that the biological characteristics of the extracellular matrix changed under different oxygen concentration conditions, leading to varying levels of calcium deposition. It was found that the interaction between oxygen concentration and the inducer influenced the expression of ALP (Browe et al., 2019). Among them, the mRNA expression in cells cultured in the three oxygen concentrations was higher in the mineralized group than in the non-mineralized group. These results suggest that ALP mRNA expression could be significantly promoted in DPCs after mineralization induction. The expression of ALP mRNA in mineralized cells under 5% oxygen concentration was higher than in mineralized cells under 21%, but higher than in those under 3%. ALP, which is mainly expressed in highly mineralized areas of bone tissue, is an enzyme that can decompose phosphate compounds and is considered a marker of early dentin formation, playing an important role during this stage (Yamamoto et al., 2007). Previous studies have found that 1% hypoxia inhibits the mineralization of human periodontal ligament cells (hPLCs), with a downregulation of ALP expression (Hsu et al., 2013). Hypoxia has also been shown to induce apoptosis and autophagy in hPLCs (Song et al., 2012). Additionally, hypoxia has been proven to promote the expression of ALP in certain types of cells (Corley et al., 2005; Fan et al., 2010; Huang et al., 2011; Li et al., 2014). Our study clarifies that this effect is oxygen-concentration dependent in DPCs, with 5% O_2_ being optimal for ALP expression and odontogenesis, while 3% O_2_ is inhibitory. This biphasic response may help reconcile some conflicting reports in the literature that often use a single hypoxic tension.

The protein levels of RUNX2, DSPP, and OCN in each group of cells were detected. It was found that the expression of odontogenic differentiation-related factors (ODRF) was enhanced by the interaction between oxygen concentration and the inducer. Among them, mRNA and protein expression in cells cultured in the three oxygen concentrations were higher in the mineralized group than in the non-mineralized group. These results suggested that the mRNA and protein levels of ODRF could be significantly promoted in DPCs after mineralization induction. The expression of ODRF in mineralized cells with 5% oxygen concentration was higher than in mineralized cells with 21% oxygen concentration and 3% oxygen concentration. These findings showed that a 5% oxygen concentration hypoxic microenvironment promoted odontogenic differentiation of DPCs, while the 3% oxygen concentration hypoxic microenvironment inhibited it. RUNX2, a transcription factor, plays a crucial role in tooth and bone development. RUNX2 knockout mice exhibit a lack of osteoblasts and bone formation, and dentin structures do not form. Thus, RUNX2 plays a significant role in dentin differentiation (Sreenath et al., 2003). RUNX2 is also associated with genetic diseases such as congenital osteogenesis defects, with RUNX2 deficiency being the cause of acromioclavicular dysplasia syndrome (Hordyjewska-Kowalczyk et al., 2019; Jung et al., 2018; Zhang et al., 2017). DSPP plays an essential role in tooth development. Sreenath et al. (Sreenath et al., 2003) created a mouse model of DSPP gene deletion and found that the pulp cavity was wider, dentin thickness increased, and mineralization decreased, similar to clinical cases of human dentin hypoplasia. Therefore, DSPP is often considered a specific marker for differentiating dental pulp stem cells into odontoblasts. OCN, also known as gamma-carboxyglutamate osteoprotegerin (Manolagas, 2020), is a specific marker of dentin and osteogenesis, belonging to the nonspecific collagen family like DSPP.

The results indicated that 5% oxygen concentration encouraged odontogenic differentiation of DPCs, while 3% oxygen concentration inhibited the process. The findings suggest that 3% hypoxia helps maintain the undifferentiated state of DPCs. Therefore, under 3% oxygen concentration, odontogenic differentiation could not be induced as effectively, and the differentiation was inhibited compared to cells cultured at 21% oxygen concentration. Consistent with the low oxygen environment within the healthy pulp cavity, we believe that DPCs remain undifferentiated in the pulp cavity. When hard tooth tissue is worn or stimulated by inflammation, the exposure of dentin tubules may lead to an increase in local oxygen concentration or other signaling cues, triggering DPC differentiation into odontoblast-like cells and the formation of secondary dentin. These results are consistent with findings that 3% hypoxia inhibits DPC differentiation into dentin (Iida et al., 2010) and Ito’s results, which indicated that 5% hypoxia promoted DPC differentiation into dentin (Ito et al., 2015). Although differences in experimental conditions make direct comparison difficult, the conclusions of this study align with their results, supporting the observed outcome, and highlighting the importance of specific oxygen thresholds.

HDAC family members that may be involved in the odontogenesis of DPCs in the hypoxic microenvironment were screened by RT-qPCR. Among them, HDAC1, 4, and 6 were the factors with statistically significant differences in mRNA expression. Epigenetic research on adult stem cells derived from the oral cavity has been increasing, with notable achievements in stomatology. The regulation of epigenetics in the process of pulp regeneration in DPCs has been studied (Duncan et al., 2016). It has been reported that HDAC1, 4, and 6 are involved in the osteogenesis of stem cells. Inhibition of HDAC1 can promote the expression of osteogenic differentiation-related genes such as osterix, osteocalcin, osteopontin, and ALP. HDAC4 is also related to osteogenesis. In osteoblasts, HDAC4 deacetylates osteocalcin, stabilizing its structure and thus promoting osteogenic differentiation (Jeon et al., 2006; Li et al., 2009; Liu et al., 2018; Lu et al., 2016; Makinistoglu and Karsenty, 2015). Studies have shown that HDAC6 specifically interacts with the carboxyl end of RUNX2, triggering its transfer from the cytoplasm to chromatin (Westendorf et al., 2002), and inhibiting the early differentiation promoter of osteoblasts by deacetylating the p21 promoter of RUNX2. HDAC family members are involved in cell proliferation, differentiation, and the cell cycle. Bioinformatics analysis of public datasets predicted that HDAC1 would play a key role in this process. It has been confirmed that HDAC1 is involved in cellular regulation mechanisms in the hypoxic microenvironment (Kim et al., 2022). Studies have demonstrated that the hypoxic microenvironment promotes the expression of HDAC1 (Lee et al., 2010; Lin et al., 2016; Tsai et al., 2016; Yoo et al., 2008). Therefore, our team speculates that DPC differentiation under hypoxia is related to HDAC1. The Western blot experiment further confirmed the relationship between HDAC1 protein levels and hypoxia and DPC differentiation.

**FIGURE 6:**
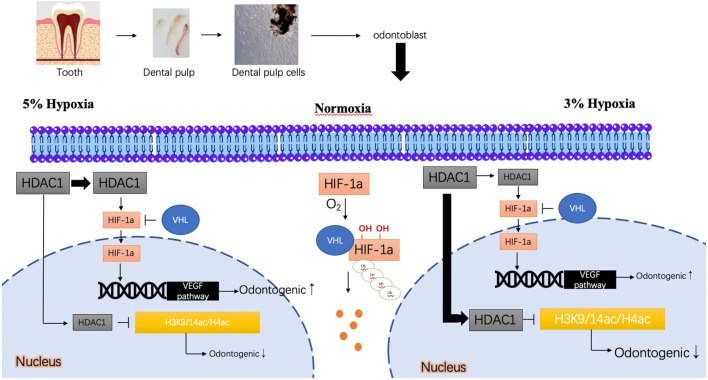
Predicted mechanism of odontogenic differentiation of DPCs under different oxygen concentrations. Under normoxia, HIF-1α is degraded. Under 5% hypoxia, HIF-1α is stabilized, potentially aided by HDAC1 activity, promoting nuclear entry and transactivation of genes like VEGFA, leading to enhanced odontogenesis. HDAC1 may also influence chromatin accessibility for odontogenic genes. Under 3% hypoxia, while HIF-1α might be stabilized, a repressive role of HDAC1 (e.g., via histone deacetylation like H3K9/14ac, H4ac at odontogenic gene promoters, shown hypothetically) may dominate, inhibiting differentiation. Inhibition of HDAC1 (e.g., by VPA) under 3% hypoxia may relieve this repression, promoting differentiation, despite potentially reducing HIF-1α levels. Conversely, under 5% hypoxia, VPA may disrupt the positive regulatory role of HDAC1 on HIF-1α or odontogenic pathways, thus reducing differentiation.

To further investigate the mechanistic roles of HIF-1α and HDAC1, we employed pharmacological inhibitors. Our study suggests that the differential outcomes of DPC odontoblastic differentiation under varying oxygen concentrations (3% vs. 5% O_2_) are, at least in part, mediated by HDAC1 and its interplay with HIF-1α. The proposed mechanism is illustrated in Figure 6. Under normoxic conditions, HIF-1α is rapidly hydroxylated by prolyl hydroxylases (PHDs) in an oxygen-dependent manner, leading to its recognition by the von Hippel-Lindau (VHL) E3 ubiquitin ligase complex, followed by ubiquitination and proteasomal degradation (Yu et al., 2001; Lee et al., 2004). Consequently, HIF-1α levels are low, and it cannot efficiently translocate to the nucleus to activate target genes. In a hypoxic microenvironment (e.g., 5% O_2_), reduced oxygen availability inhibits PHD activity, stabilizing HIF-1α, allowing it to accumulate, enter the nucleus, and activate target genes like VEGF, which can promote odontogenesis (Lee and Kim, 2012; Kim et al., 2007). Our results suggest that HDAC1 may further contribute to HIF-1α regulation. Inhibition of HDACs by VPA led to decreased HIF-1α protein levels, particularly under 5% hypoxia, suggesting HDAC activity is required for maximal HIF-1α accumulation or stability under these conditions. Conversely, under severe hypoxia (3% O_2_), where differentiation was inhibited, VPA treatment rescued differentiation. This suggests that at 3% O_2_, HDAC1 (and possibly other HDACs inhibited by VPA) might exert a dominant repressive effect on odontogenic genes, potentially through histone deacetylation. Thus, HDAC1 could have a dual role: promoting odontogenesis at 5% O_2_ possibly via HIF-1α related pathways, while inhibiting it at 3% O_2_ potentially through direct epigenetic repression of differentiation genes by deacetylating histones, thereby altering chromatin accessibility for transcription factors crucial for odontogenesis. When VPA inhibits HDAC1, it may prevent HIF-1α stabilization (seen as reduced HIF-1α protein) but simultaneously relieve the repressive deacetylation of odontogenic gene promoters, leading to a net effect that depends on the specific oxygen context. This complex interplay results in distinct differentiation outcomes: 3% hypoxia inhibits odontogenic differentiation (rescued by VPA), while 5% hypoxia promotes it (attenuated by VPA and Oltipraz).

The clinical relevance of these findings lies in the potential to optimize DPSC-based regenerative therapies. Understanding the biphasic response to oxygen could inform the design of scaffolds that establish specific oxygen microenvironments or guide preconditioning strategies for DPSCs. Furthermore, targeting the HDAC/HIF-1α axis, perhaps with selective HDAC inhibitors, could offer a pharmacological approach to enhance pulp regeneration. For instance, transient HDAC inhibition might be beneficial in scenarios where severe hypoxia limits differentiation, or careful modulation of HIF-1α could steer DPSC fate. However, translating these findings to clinical practice faces challenges, including the precise control of local oxygen tension *in vivo*, the specificity and potential side effects of HDAC inhibitors (Gordon et al., 2015), and the need for targeted delivery systems.

### Limitations of the study

This study has several limitations. Firstly, Valproic acid (VPA) is a broad-spectrum HDAC inhibitor affecting multiple Class I and IIa HDACs, not solely HDAC1. While our mRNA screening highlighted HDAC1, 4, and 6, the specific contribution of HDAC1 versus other VPA-sensitive HDACs to the observed effects cannot be definitively concluded without more specific interventions like siRNA-mediated knockdown or highly selective inhibitors. Secondly, the claim that HDAC1 modulates HIF-1α stability was inferred from changes in HIF-1α protein levels upon VPA treatment; direct assays of protein stability (e.g., cycloheximide chase experiments) were not performed. The observed changes could also involve transcriptional or translational regulation. Thirdly, the interaction between HDAC1 and HIF-1α was predicted bioinformatically using public databases and not experimentally validated in our system (e.g., via co-immunoprecipitation). Finally, while we infer effects on chromatin remodeling based on HDAC inhibition, direct measurements of histone acetylation marks (e.g., H3K9ac, H3K14ac, H4ac as depicted in our hypothetical model Figure 6) at specific gene promoters were not conducted. Future studies incorporating these experimental approaches would provide more definitive mechanistic insights.

## Conclusion

In summary, a 3% hypoxic environment promoted early cell cycle progression (S/G2/M increase at 48 h) but inhibited odontogenic differentiation and longer-term proliferation, while a 5% hypoxic environment supported robust proliferation and promoted odontogenesis. HDAC1 appears to play a crucial regulatory role in the odontogenesis of DPCs in a hypoxic microenvironment, partially by participating in the HIF-1α signaling pathway and putatively by regulating chromatin structure through its deacetylase activity. Further studies are needed to better understand the complete mechanism of HDAC1 regulation in this complex process, to dissect the specific roles of different HDAC isoforms, and to confirm the direct nature of the proposed molecular interactions and chromatin modifications in DPCs at different stages of tooth development and regeneration.

## Data Availability

The original contributions presented in the study are included in the article/Supplementary Material, further inquiries can be directed to the corresponding authors.

## Abbreviations

DPCs, dental pulp cells; hDPCs, human dental pulp cells; DPSCs, dental pulp stem cells; MSCs, mesenchymal stem cells; DSPP, dentin sialophosphoprotein; RUNX2, runt-related transcription factor 2; ALP, alkaline phosphatase; OCN, osteocalcin; HDAC, histone deacetylase; HIF-1α, Hypoxia Inducible Factor 1, Alpha Subunit; VPA, Valproic acid; VEGFA, vascular endothelial growth factor A; DMEM, Dulbecco’s modified Eagle’s medium; FBS, fetal bovine serum; PI, propidium iodide; CCK-8, Cell Counting Kit-8; ARS, Alizarin Red S; MI, mineralization induction; GEO, Gene Expression Omnibus; GSEA, Gene Set Enrichment Analysis; KEGG, Kyoto Encyclopedia of Genes and Genomes; GO, Gene Ontology; DAVID, Database for Annotation, Visualization and Integrated Discovery; VHL, von Hippel-Lindau; PHD, prolyl hydroxylase domain protein.
