# Supplementary figures and images for: Oxygen concentration modulates HDAC1-Mediated regulation of osteogenic signaling pathways in dental pulp cells
Source: Front Cell Dev Biol. 2025 Sep 17;13:1627763. doi: 10.3389/fcell.2025.1627763 (PMC12485506; doi:10.3389/fcell.2025.1627763)

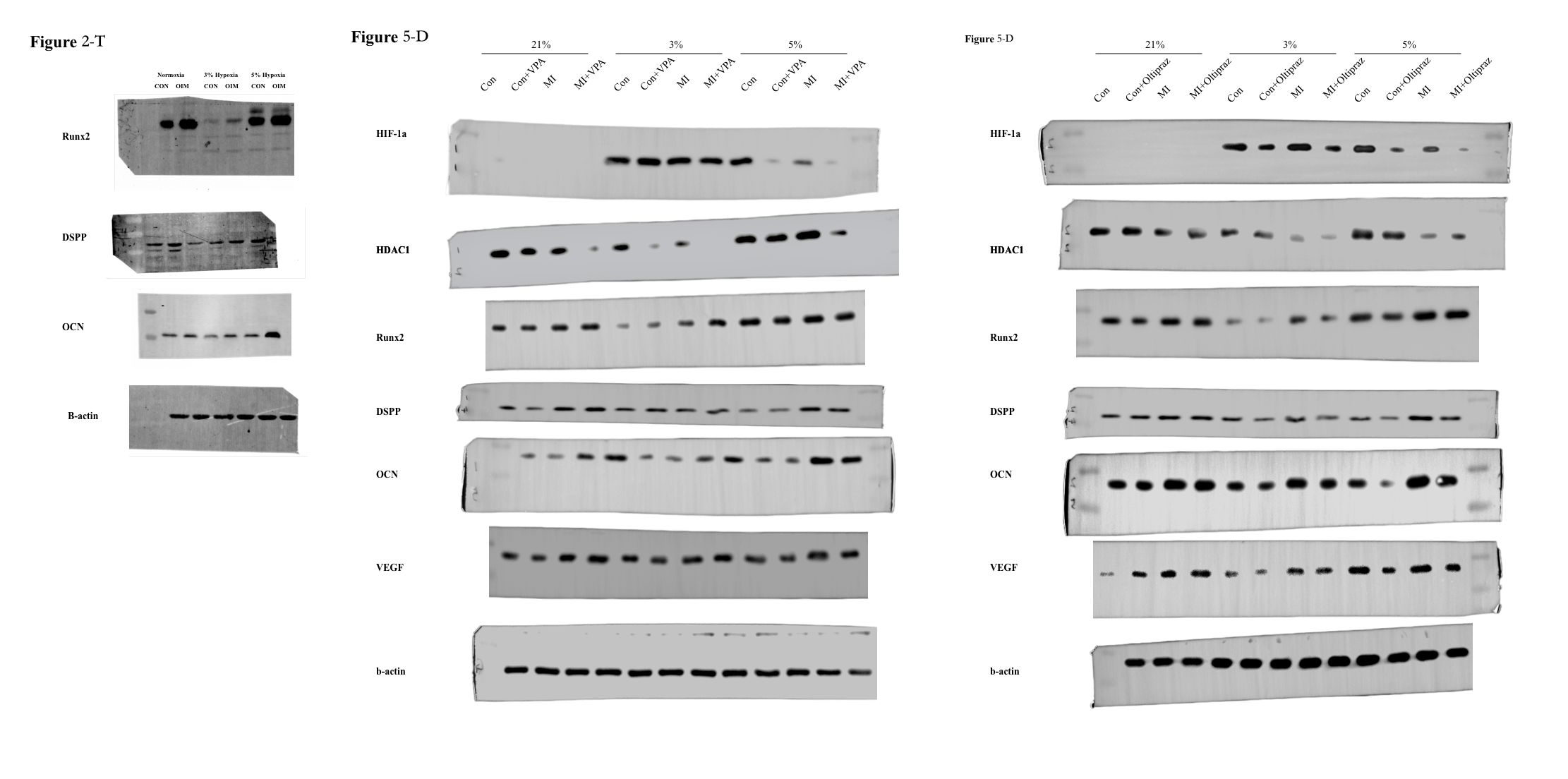

Supplement: Supplementary file 1 [file Image1.png]
